# Recent advances in the link between physical activity, sedentary behavior, physical fitness, and colorectal cancer

**DOI:** 10.12688/f1000research.9795.1

**Published:** 2017-03-01

**Authors:** Vikneswaran Namasivayam, Sam Lim

**Affiliations:** 1 Department of Gastroenterology and Hepatology, Singapore General Hospital, Singapore, Singapore; 2 Duke NUS Medical School, Singapore, Singapore; 3 Respiratory Inflammation and General Medicine of AstraZeneca Global Medicine Development Centre, Shanghai, China; 4 Office of Clinical Science, Duke-NUS Medical School, Singapore, Singapore

**Keywords:** colorectal cancer, sedentary behaviour, cardiorespiratory fitness, risk factor

## Abstract

Physical inactivity is a well-established risk factor for colorectal cancer (CRC). Recent studies have characterized physical activity (PA), sedentary behavior, and cardiorespiratory fitness as distinct, interrelated constructs that influence the risk of CRC and related outcomes. PA levels required to confer protection against CRC may be higher than previously thought. Sedentary behavior, defined as time spent sitting, increases CRC risk independent of PA and may require novel interventions distinct from those targeting PA. Finally, cardiorespiratory fitness is inversely associated with CRC risk and mortality and may provide a potential tool for risk stratification and intervention.

## Introduction

A consistent body of literature demonstrates a protective role for physical activity (PA) against colon cancer. Up to a third of common cancers and a fifth of colorectal cancers (CRCs) in industrialized nations have been ascribed to excess weight and insufficient PA, and the disease burden attributable to inactivity is expected to rise in developing countries as well ^1– 7^. In addition to counteracting obesity, PA confers beneficial effects against CRC through other pathways, and physical inactivity is a risk factor for CRC that is independent of obesity. Almost a third of adults worldwide are currently inactive, and the trend toward physical inactivity starts in early life—a reflection of modern-day living, working, and commuting being carried out in an environment designed to avoid physical labor ^8^. It is now recognized that a sedentary lifestyle comprises three distinct but interrelated concepts: PA, sedentary behavior, and cardiorespiratory fitness (CRF). This review describes recent developments in our understanding of how each of these factors impacts on CRC risk.

## Physical activity, sedentary behavior, and cardiorespiratory fitness

PA may be defined as any bodily movement produced by skeletal muscles that result in energy expenditure ^9^. It is characterized by the following dimensions—frequency, duration, and intensity—and may be accumulated within the following domains: recreation, occupation, transportation, and domestic. PA levels can be measured by self-report through logs or questionnaires. PA increasingly is being assessed via objective measures such as accelerometers, which are wearable electronic devices that record the volume, intensity, and frequency of activity. PA is often quantified as hours of activity per week or as metabolic equivalent (MET)-hours per week. MET is a physiological measure expressing the energy cost of physical activities where 1 MET is the average resting energy expenditure of humans seated at rest. Moderate-to-vigorous physical activities are those that entail an energy expenditure of 3 to 8 METs, whereas light-intensity activity behaviors usually require an expenditure of less than 3 METs ^10^. The MET score for various physical activities has been estimated by researchers and reported within the Compendium of Physical Activities ^10^. The Compendium is frequently used to estimate the energy expenditure of physical activities reported within epidemiologic studies ^10^.

Existing research and public health recommendations have placed an emphasis on increasing PA that fall within the moderate to vigorous intensity of the PA spectrum. However, there is growing recognition that light-intensity activities constitute a significant portion of daily energy expenditure with attendant health benefits ^11, 12^.

Sedentary behavior refers to any waking behavior characterized by an energy expenditure of not more than 1.5 METs while in a sitting or reclining posture ^13^. This includes activities such as sitting, watching TV, using the computer or other screen-based entertainment, and spending time in automobiles. Sedentary behavior is not merely inadequate amounts of moderate-to-vigorous PA but a behavioral entity that may have distinct physiological effects ^14^. Sedentary behavior may vary among physically active populations. Physically active individuals who satisfy expert recommendations for moderate-to-vigorous PA may still be sedentary for their remaining waking hours and thus are at risk of detrimental health outcomes ^12, 15^. The importance of sedentary behavior stems from the risk it confers on longevity, cancer and other chronic diseases that are independent of PA, the growing epidemic of sedentary behavior that typifies urban living and the recognition that sedentary behavior may require different interventions that are not adequately addressed by current public health promotion measures targeting PA ^12, 16– 19^.

CRF is the capacity to use atmospheric oxygen for cellular energy production via aerobic metabolism. CRF is defined on the basis of maximal oxygen intake or maximal work capacity measured by incremental exercise testing with a cycle ergometer or treadmill. The gold-standard measure of CRF is the maximum oxygen uptake (VO _2_ max) (measured in liters per minute) and is a reflection of the patient’s functional aerobic capacity. The term physical activity and fitness are sometimes used interchangeably but refer to distinct concepts. PA characterizes a behavior whereas CRF describes the capacity to achieve a certain performance level ^9^. Although there is a significant inheritable component to VO _2_ max (up to 50% in sedentary adults), PA is considered the primary determinant of CRF ^19– 21^. Although CRF is modestly correlated with PA, both provide distinct information. Unlike PA and sedentary behavior, which are estimated by questionnaires, CRF is measured by incremental exercise testing and provides an objective, quantitative, and highly reproducible measure of the functional consequences of PA which may be used for risk stratification and serve as a potential target for intervention.

## Physical activity and colorectal cancer

Regular PA is protective against colon cancer. PA reduces the risk of colon cancer by approximately 20%–25% among both men and women in a dose-response manner ^20, 21^. The protective effect has been consistently found in studies of various designs, diverse populations, in subjects of varying body mass index (BMI), across various domains of PA, and after statistical control for various lifestyle factors, indicating that the relationship is unlikely to be due to confounding health behaviors ^22– 26^. The protection conferred by PA appears to be similar in the proximal and distal colon ^27, 28^, and conflicting results in earlier studies probably reflect small sample sizes and differing definitions of proximal and distal colon.

PA in the context of an overall healthy lifestyle characterized by healthy diet, low alcohol consumption, no smoking, PA, healthy BMI, and adequate sleep confers protection against colon cancer, but the relationship is inconsistent for rectal cancer ^29, 30^. The evidence linking PA with rectal cancer, in contrast to that with colon cancer, is conflicting, although a recent meta-analysis indicates a protective effect of leisure-time PA on the risk of rectal cancer ^31– 33^.

PA is also associated with an estimated 15% reduction in the risk of colonic adenoma, the precursors to carcinoma. The association holds for both genders and is stronger for advanced adenoma (35% risk reduction) ^34, 35^. The risk of recurrent adenoma, in contrast to that of incident adenomas, appears to be reduced with PA only in men but the duration of follow-up studied was relatively short ^36, 37^. Unlike the case for adenoma, a protective effect for PA on serrated polyps, which represent an alternate pathway to colon carcinogenesis, remains to be established ^38– 40^.

PA, before and after a diagnosis of CRC, also reduces the risk of all-cause mortality and CRC-specific mortality in a dose-dependent manner ^41, 42^. Every 15 MET-hours per week increase in PA – approximately equivalent to walking for 5 hours per week - after a diagnosis of CRC reduces total mortality by 38% and CRC-specific mortality by 35% respectively. Furthermore, *increasing* PA levels following a diagnosis of cancer was associated with a decreased risk of total mortality ^41^. The conclusions of the meta-analysis may be affected by reverse causality resulting from inclusion of cancer patients who had lower PA levels due to symptoms of the disease at the time of PA assessment. Randomized controlled trials are ultimately required to establish causation and determine the true effect of PA as an intervention.

Emerging studies have shed further light on the optimum exercise dose (that is, frequency, duration, and intensity) and the relative benefits of aerobic PA versus resistance PA. Studies suggest that the level of PA required to reduce overall mortality risk may be higher than current public health recommendations ^43, 44^. In a cohort study of over 43,000 US male health professions, PA was inversely associated with risk of digestive system cancers (hazard ratio (HR) 0.74 for ≥63.0 versus ≤8.9 MET-hours per week, 95% confidence interval (CI) 0.59–0.93). Aerobic exercise was especially beneficial compared with resistance exercise, and optimum benefit was derived at 30 MET-hours per week (HR 0.68, 95% CI 0.56–0.83), which translates to approximately 10 hours of walking per week at average pace ^45^. The benefit was similar irrespectively of intensity of PA so long as the MET score was achieved. A similar level of PA has been associated with reduced mortality in patients with CRC ^44^. This contrasts with existing American Cancer Society recommendations of at least 150 minutes of moderate-intensity or 75 minutes of vigorous-intensity activity each week, or an equivalent combination, preferably spread throughout the week ^43^. Although there is a dose-response association between PA and colon cancer, which suggest benefits even with a little additional PA, higher levels of PA may be required to attain optimal benefits in CRC prevention.

## Sedentary behavior and colorectal cancer

Sedentary behavior is increasingly recognized as a risk factor for CRC incidence and mortality independent of PA ^44, 46– 49^. Sedentary behavior as characterized by time spent watching TV, occupational sitting time, and total sitting time was associated with a 54%, 24%, and 24% increased risk of colon cancer, respectively, in a meta-analysis ^46^. Sedentary behavior is also associated with an increased risk of all-cause mortality, CRC-specific mortality ^44, 50^, and lower quality of life in CRC survivors ^51^.

Prolonged TV viewing is associated with an increase in the risk of colorectal adenoma independent of leisure time PA, particularly for high-risk adenoma, in a study of male health professionals, suggesting that sedentary behavior potentially acts early in carcinogenesis ^52^. Sedentary behavior is also associated with a higher risk of colorectal adenoma recurrence among men but not women ^37^.

It is unclear whether the deleterious effects of prolonged sitting on CRC can be overcome by simply increasing PA levels. A recent meta-analysis of over 1 million subjects studied the association of PA and sedentary behavior with all-cause mortality and demonstrated that high levels of moderate-intensity PA (60–75 minutes daily) are required to eliminate the risk of all-cause mortality associated with a sedentary lifestyle ^53^. However, this level of activity decreases, but does not eliminate, the risks of all-cause mortality associated with prolonged TV viewing. It is unknown whether this applies to the risk of CRC as well.

There remain significant challenges in translating the current understanding of the impact of sedentary behavior on CRC into interventions with meaningful clinical impact.

Although sedentary behavior has been linked with various diseases, there are limited data on the minimum reduction in sedentary behavior required to give rise to health benefits. Current guidelines that advocate limiting sedentary behavior do not offer quantitative recommendations or specific strategies to reduce sitting time ^43^. Sedentary behavior may also require specific interventions, targeted at interrupting extended sitting with frequent short activity breaks, that are distinct from those designed to increase PA ^54^. Interventions focused on increasing PA levels do not have a consistent impact on reducing sedentary behavior ^55, 56^. Current interventions targeting sedentary behavior have a modest impact on reducing sitting time and their impact on health outcomes remains to be determined ^56, 57^. Assessment methods for sedentary behavior also need to be standardized and validated. Subjective measures based on self-report are limited by measurement error, whereas more objective measures are costly and lack information on specific domains of sedentary behavior ^58^. Existing studies linking sedentary behavior to CRC have focused largely on Caucasian males, and findings need to be corroborated in more diverse populations ^59^.

## Cardiorespiratory fitness and colorectal cancer

CRF is associated with a decreased risk of CRC incidence and mortality. In a prospective cohort study of almost 14,000 community-dwelling men, high CRF at midlife was associated with a 44% reduction in the risk of CRC compared with low CRF. Every 1-MET increase in CRF was associated with a 9% relative risk reduction in CRC risk. In addition, CRF in midlife was associated with a decreased risk of death from cancer or cardiovascular disease following a diagnosis of lung, colorectal, or prostate cancer in men, suggesting a sustained benefit of fitness into old age even in the setting of a cancer diagnosis ^60^.

These findings expand on earlier studies demonstrating a protective effect of CRF ^59– 61^. In an earlier study of over 38,000 men followed up for 29 years, CRF was inversely associated with the risk of total digestive cancer mortality, and men in the moderate and high CRF groups showed 34% and 44% lower risk, respectively, of dying of digestive cancers. Men with an exercise capacity of less than 8 METs had a threefold higher risk of dying of digestive cancer compared with those with higher MET level (≥11). Being fit (the upper 80% of CRF) was also associated with a lower risk of mortality from colon cancer (HR 0.61, 95% CI 0.37–1.00) and CRC (HR 0.58, 95% CI 0.37–0.92) compared with being unfit (the lowest 20% of CRF) ^61^.

In an earlier cohort study of 21,000 men with pre-diabetes and diabetes, moderate fitness was associated with a 29%–47% reduction and high fitness was associated with 24%–56% reduction in the risks of cancer mortality. Among all men, being fit was associated with a 45% lower risk of mortality from gastrointestinal cancer and 47% risk of CRC ^62^. These findings are consistent with those of a study of Finnish men, which demonstrated that good CRF was associated with decreased cancer incidence and mortality ^63^.

These findings indicate that CRF, which provides a more objective and reproducible measure of the functional consequence of PA, confers beneficial effects on CRC incidence and mortality ^20^. In addition, the decreased risk of death from cancer or cardiovascular disease following a CRC diagnosis may relate to the significant burden of cardiac and metabolic diseases present in CRC patients that stems from sharing common risk factors ^64^. Improvements in CRC management may increase the contribution of heart disease as a competing cause of mortality in CRC survivors and thus account for part of the beneficial effects of CRF on mortality following a diagnosis of CRC. Also, increased CRF potentially leads to better tolerance and completion of surgery and adjuvant treatment for CRC, thus leading to improved survival ^65, 66^.

These studies raise the potential for improving cancer-related outcomes through CRF-based risk stratification and exercise-based interventions targeting an improvement in CRF. However, several challenges need to be overcome. Existing studies have focused largely on men and these findings need to be replicated in studies on women. Future studies need to move beyond studying disease associations to demonstrate whether CRF actually improves discrimination of risk of CRC-related outcomes and improves classification of risk profiles for individual subjects. Current cohort studies have measured CRF at a single time point, given the logistic challenges of conducting exercise testing on a large scale. Disease associations have also been defined in relation to the lowest category of CRF as the reference point. It is unclear what target CRF or minimum improvement in CRF over time must be attained and sustained through interventions to achieve an improvement in CRC-related outcomes, if any. There are currently no published randomized controlled trials to confirm that PA lowers the risk of CRC in the general population or CRC recurrence or mortality among CRC survivors. The ongoing CHALLENGE (Colon Health and Lifelong Exercise Change) trial will provide further insight into whether PA will improve outcomes in colon cancer ^67^. Future studies may aim to use CRF measures as a potential means to titrate the dosimetry of exercise regimens to achieve increments in CRF needed to improve CRC outcomes.

## Conclusions

Our understanding of the link between physical inactivity and CRC has expanded to recognize PA, sedentary time and CRF as distinct, interrelated concepts which impact on CRC incidence and outcomes. PA levels required to confer protection against CRC may be higher than previously thought. Sedentary behavior is now recognized to have deleterious effects on CRC independent of PA and may require distinct and novel interventions from those targeting PA. CRF is associated with CRC risk and mortality and may provide a potential tool for risk stratification and intervention. Future studies should focus on translating current knowledge into interventions that improve health outcomes.

## Abbreviations

BMI, body mass index; CI, confidence interval; CRC, colorectal cancer; CRF, cardiorespiratory fitness; HR, hazard ratio; MET, metabolic equivalent; PA, physical activity; VO _2_ max, maximum oxygen uptake.
